# Factors that influence scope of practice of the chiropractic profession in Australia: a scoping review

**DOI:** 10.1186/s12998-022-00428-2

**Published:** 2022-04-14

**Authors:** Desmond Wiggins, Aron Downie, Roger Engel, Benjamin T. Brown

**Affiliations:** Department of Chiropractic, Macquarie University, Sydney, Australia

**Keywords:** Chiropractic, Scope of practice, Australia, Scoping review

## Abstract

**Introduction:**

The World Health Organization describes chiropractic as a health profession that treats the musculoskeletal system and the effects of that system on the function of the nervous system and general health. Notwithstanding such descriptions, scope of practice remains a contentious issue in Australia chiropractic with various authors defining it differently. To date, the peak governing body, the Chiropractic Board of Australia, has focused on title protection rather than defining a scope of practice for the profession. A well-defined scope of practice is important, as it helps to identify what is acceptable in the profession and the role chiropractic has in the broader healthcare system.

**Objective:**

The objective of this scoping review was to explore the literature on the factors that influence scope of practice of chiropractic in Australia.

**Methods:**

This study employed scoping review methodology to document the current state of the literature on factors that influence scope of practice of the chiropractic profession in Australia.

**Results:**

A total of 1270 articles were identified from the literature search. Six studies fulfilled the inclusion criteria and were included in the final analysis. Four factors that influence scope of practice were identified: education, professional identity, patient safety, and organisational structure.

**Conclusion:**

The results of this study will inform future discussions around establishing a framework for a more comprehensive scope of practice for the chiropractic profession in Australia. Such a framework has the potential to benefit patient safety, professional identity, public perception, education, and regulation of the profession.

**Supplementary Information:**

The online version contains supplementary material available at 10.1186/s12998-022-00428-2.

## Introduction

Recent estimates highlight a global shortage of healthcare professionals [1] including in the field of musculoskeletal (MSK) medicine [2]. Musculoskeletal conditions are the primary cause of disability and the number one contributor to years lived with disability worldwide [3, 4]. In light of this, allied health professions that provide care for MSK conditions, such as chiropractic, have a role to play in preventing disability and reducing the burden of disease related to these conditions [2, 5].

In Australia, back and neck pain cause significant disability and loss of productivity and account for more than 21 million patient visits per year [6]. This represents a considerable portion of the country’s total healthcare expenditure, part of which is spent on chiropractic services [7–9]. Chiropractic is the eleventh largest (out of sixteen) regulated health care professions in Australia with 5582 practitioners as of September 2021 [10]. While the profession contributes to various levels of the Australian healthcare system, there is little that delineates the magnitude or extent of those contributions.

The World Health Organization (WHO) describes chiropractic as a health profession that treats the musculoskeletal system and the effect it has on the function of the nervous system and general health [11]. Notwithstanding such definitions, debate has continued in Australia about scope of practice of the profession [12] with various authors defining it differently [13].

‘Scope of practice’ comprises three separate, but interrelated levels: ‘jurisdictional’ (legislative/regulatory) [14–16], ‘professional’ (the profession) [14, 15, 17]; and ‘personal’ (individual practitioner) [14, 18–20]. Jurisdictional scope of practice is founded on Government practice acts that contain regulations to ensure patient safety [14, 16, 21]. Professional scope of practice is grounded in a unique body of evidence, supported by educational preparation, and linked to an existing or emerging practice framework [14]. Moreover, it is based on the rules, regulations and boundaries that convey the role of the profession and provide protection to the public [17, 18]. Personal scope of practice is centred on the activities that an individual health care practitioner is educated and trained for, and that they can perform [14] in a way that does not pose any danger to the public or themselves [15, 17]. Currently, personal scope of practice is more clearly defined than professional scope of practice [20].

Despite having the authority to develop a single scope of practice for the chiropractic profession in Australia [22], the peak regulatory authority, the Chiropractic Board of Australia (CBA), has focused on title protection rather than clearly defining scope of practice for the profession [23]. A well-defined scope of practice in a health profession is important, as it helps to identify what is acceptable in that profession and the role it has in the broader healthcare system [24–26]. A clearly defined scope of practice for the chiropractic profession would be valuable to a variety of stakeholders including patients, health care providers, professional associations and policymakers [27].

As innovative technology and new treatment modalities emerge, the importance of understanding scope of practice is increasing for professions as they are called upon to navigate the increasingly complex realm of patient care [28]. This is true for chiropractic. We recognise that a broader picture of scope of practice exists within health professions in Australia (e.g. nursing and midwifery and physiotherapy), but the objective of this scoping review is to explore the literature on the factors that influence scope of practice of chiropractic in Australia.

## Methods

Scoping review methodology was chosen as the most appropriate way to collect and organise relevant information to address our broad research question and provide a thorough examination of the existing literature. Unlike other reviews (e.g. systematic reviews), that typically focus on specific questions, scoping reviews provide an overview of the developing evidence when it is uncertain what specific questions can be posed for evidence synthesis [29].

The review has been reported against the Preferred Reporting Items for Systematic Reviews and Meta-Analyses Extension for Scoping Reviews (PRISMA-ScR) checklist. The 5-step framework of Arksey and O’Malley [30], as well as additional recommendations for conducting and reporting scoping reviews were used [31–33]. Arksey and O’Malley refined Mays’ definition of a scoping review which focused on mapping the key concepts and types of evidence underpinning a research area. A scoping review could be undertaken as a standalone project, especially where an area is complex or has not been previously comprehensively reviewed [34]. Their completed framework consists of five pivotal stages for conducting this type of review.

### Step 1: Identifying the research question

Our scoping review was guided by the following broad research question: what is known about the factors that influence scope of practice of chiropractic in Australia?

### Step 2: Identifying relevant studies

The search strategy was developed in collaboration with a university librarian and conducted using the following databases: AMED (Allied and Complementary Medicine Database), CINAHL (Cumulative Index to Nursing and Allied Health Literature), Cochrane Library, EMBASE (*Excerpta Medica* Database**)**, ICL (Index to Chiropractic Literature), MANTIS (Manual, Alternative and Natural Therapy Index System) MEDLINE, PubMed and SCOPUS. The search included all articles published between January 2011 and July 2021. This date range was selected as Australia introduced the National Registration and Accreditation Scheme (the National Scheme), to regulate practitioners of ten health professions, including chiropractic, in late 2010. Thus, 2011 was the first year dialogue around a national scope of practice could be undertaken [35].

Additional data sources were searched from Google and ProQuest. A combination of indexing terms (MeSH and non-MeSH) relevant to the research theme were used: scope AND practice AND chiropractic OR chiropractor AND Australia AND determinant/s OR barrier/s OR enabler/s OR influencer/s OR facilitator/s. (For the purpose of this study these latter terms are used synonymously). An example of the search strategy for the PubMed and CINAHL databases is included in Additional file 1: Appendix 1. Forward/reverse citation tracking was also undertaken on eligible studies. All citations were imported into Endnote (version X9.3.3, Clarivate Analytics^©^, Boston MA, USA).

### Step 3: Study selection

#### Inclusion and exclusion criteria

The review used the Joanna Briggs Institute’s (JBI) Population, Concept, Context framework for scoping reviews (PCC) [35] to align the study selection with the research question (Table 1). To be included in the study, articles had to be published in English and related to factors that influence scope of practice of chiropractic in Australia. Articles were excluded if they related to another country, referred to another regulated (e.g. pharmacy) or unregulated (e.g. massage therapy) health profession, were based on cadaveric or animal studies, or were related to topics not pertinent to scope of practice.

**Table 1:** Joanna Briggs Institute PCC framework

P—Population	Chiropractic profession in Australia
C—Concept	Factors that influence scope of practice
C—Context	Chiropractic practice in Australia

#### Screening and agreement

Two reviewers (DW, BB) independently screened the search results. Initially, records were screened by title and abstract to identify relevant, possibly relevant and irrelevant citations. As grey literature does not typically contain an abstract [36], vetting of these results was conducted using the full text version of the citation. In keeping with scoping review methodology [37], the number of studies included and excluded at each stage, along with the reasons for exclusion, were presented in a PRISMA flow diagram.

### Step 4: Charting the data

To collate information from the relevant literature, a Word® table (Additional file 2: Appendix 2) was created that included the following items: category; year of publication; author/s; country of origin; aim/purpose; method/study design; and factors that influence scope of practice. Data extraction was completed independently by two investigators (DW, RE).

### Step 5: Collating, summarising, and reporting results

An analysis was made of the research results including the number of papers included in the analysis, year of publication, and study design. Each eligible article was assessed for potential factors that influence scope of practice of chiropractic. During the assessment process, if information relating to factors that influence scope of practice was not available, the study was rated as unsuitable and excluded.

## Results

**Fig. 1:**
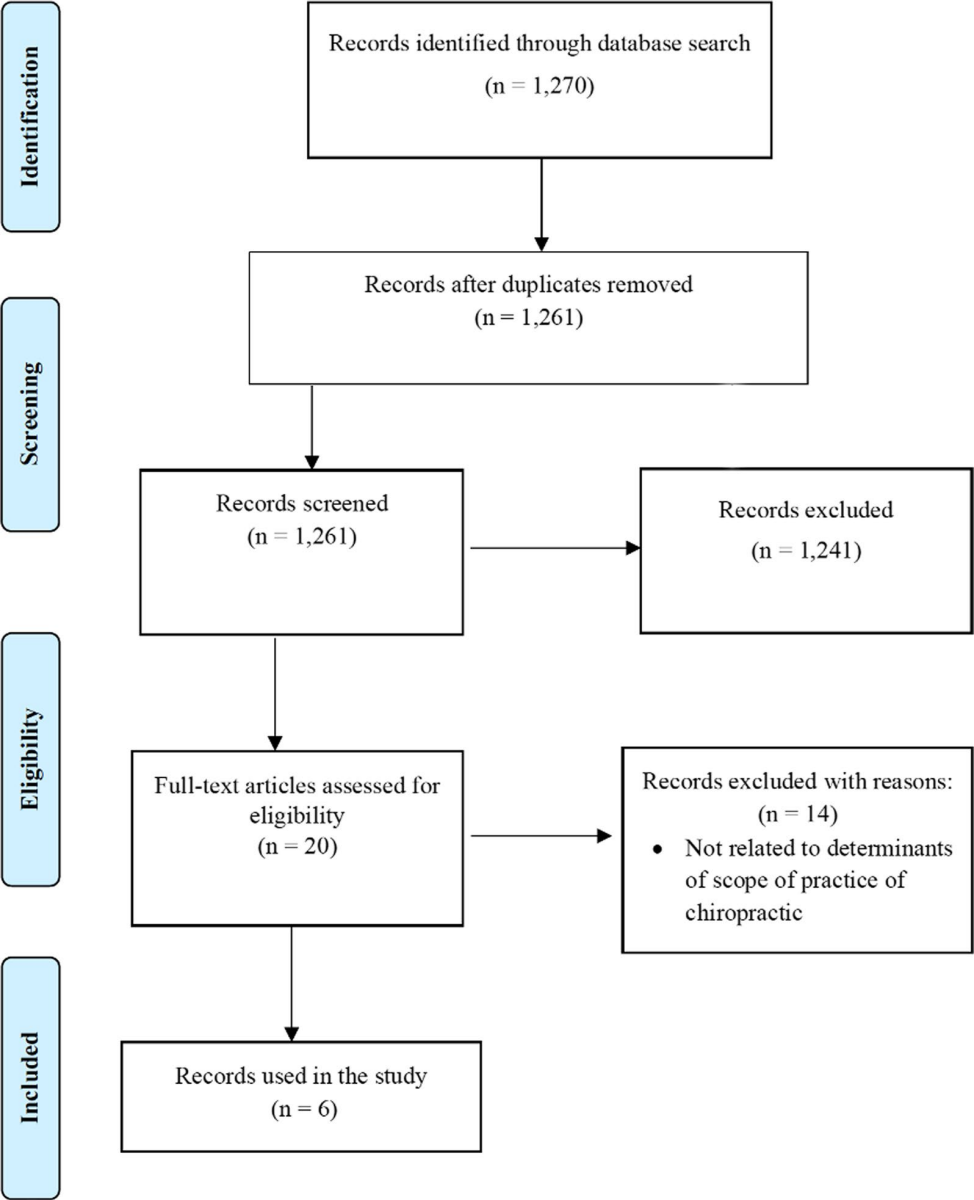
PRISMA flowchart diagram

We did not determine a priori what the factors were, rather we considered what the authors of included studies found to influence the scope of practice, then distilled these into the key factors used in our review. A total of 1270 articles were identified from the literature search. Following the removal of duplicates, 1261 papers were screened, of which 1241 articles were excluded. The full text of 20 studies were then assessed for eligibility (see PRISMA flow chart—Fig. 1). Fourteen studies were excluded based on lack of reporting factors that influence scope of practice. Six studies were included in the final analysis. No additional articles were discovered following forward/reverse citation tracking. The six studies consisted of one scoping review, one qualitative study, two cross-sectional studies, and two news articles. Within the studies we found, the factors were not always classified as either jurisdictional, professional, or personal scopes, but where an author mentioned a specific scope, it was mentioned in this review.

### Reported factors that influence scope of practice

Four factors were reported in the literature that influenced scope of practice of chiropractic in Australia:

#### Education

Two of the included studies reported that chiropractic education was an influencing factor on scope of practice. For example, de Luca et al. [38] surveyed students in chiropractic programs in Australia and New Zealand about their views on several elements within chiropractic including scope of practice, as it relates to chiropractic education. The authors reported that a divergence existed between individual chiropractic educational institutions regarding scope of practice. Despite de Luca et al. [38] not describing a defininitve reason for the variance, they suggested a combination of the chiropractic institution and the level of education before chiropractic study, may explain much of the variance. Similarly, Innes et al. [39] found chiropractic education was a “focal element influencing the scope of practice of chiropractic in Australia”.

#### Professional identity

One study undertook a cross-sectional survey of graduates from Australian and New Zealand chiropractic universities [38]. The study found that Australian graduates had divergent opinions about what comprised professional identity depending on the institution they had attended. For example, approximately 75% of respondents felt that chiropractors should be considered complementary/alternative health care practitioners, while the remainder stated they should be viewed as allied health care practitioners [38].

Two news articles, McArthur [25] and ABC Premium News [40], identified patient safety as a factor that influenced scope of practice, particularly when SMT was being utilised in paediatric populations. An Australian study by Engel et al. [41] found that GPs views about scope of practice of chiropractors was influenced by a perceived notion that chiropractic is unsafe. As a result, GPs reported hesitancy in referring patients to chiropractors.

#### Organisational structure

One study found that an organisational structure could influence scope of practice of chiropractic. Netto et al. [42] reported that 78% of Fast Jet Aircrew in the Royal Australian Air Force (RAAF) experienced flight-related neck pain during or after a flight. Despite some participants reporting that chiropractic was effective, physicians within the structure were hesitant to refer patients for chiropractic treatment, as it was not part of the standard on-base healthcare services [42].

## Discussion

The objective of this study was to explore the literature on the factors that influence scope of practice of chiropractic in Australia. Four factors were highlighted by the authors of the retrieved articles: education, professional identity, patient safety and organisational structure.

### Education

Chiropractic education in Australia has two components: pre-and post-professional. Pre-professional training involves a 5-year program at one of four universities accredited by the Council on Chiropractic Education Australasia (CCEA) [43]. Training involves studies in anatomy, neuroanatomy, physiology, microbiology, histology, pathology, diagnosis, and management, including joint manipulation, of MSK conditions of the spine and extremities. In keeping with government and CCEA guidelines, protection of the public is prominent in Australian chiropractic educational programs [44]. Our study highlights that dissimilar or diverse teachings among educational institutions impact a students’ attitudes and ideologies regarding scope of practice [38].

For example, despite receiving a mostly science-based education, Australian chiropractic students were divided on the issue of whether the historical rationale for chiropractic intervention was as important as ‘science-based’ clinical reasoning [39]. Innes et al. [39] propose that the heterogeneity of teachings between chiropractic educational institutions raises two important questions. First, who decides what is chiropractic and its attendant scope of practice? Second, what is the best model to deliver the most relevant education for those seeking to become chiropractors in the twenty-first century [39]?

We could find no definitive mechanism as to how education interacts with scope of practice. de Luca et al. [38] argued that it may be the result of a combination of chiropractic institution and level of education before commencing chiropractic study. It is possible that the of scope of practice is influenced by (1) content from both delivered and hidden curricula with pre- and post graduate education [39]; (2) guidance (or lack of thereof) provided by the accreditation bodies (e.g. CCEA) in relation to graduate capabilities [44]; and more broadly, (3) an education institiution’s health care ideology may influence (or support) student views on future scope of practice [45]. This is a complex area that requires exploration in a future study.

In Australia, post-professional training falls under the heading of ‘continuing professional development’ (CPD). To maintain registration, a chiropractor is required to complete a minimum number of CPD activities or hours that fulfil the following goals:

- Seek to improve patient health outcomes, safety and experiences;
- Draw on best available evidence, including well-established and accepted knowledge that is supported by research where possible, to inform good practice and decision-making;
- Contribute directly to maintaining and improving competence (performance and behaviour) and keeping up to date in the chosen field or setting of practice; and
- Build on the existing knowledge [46].

In 2019, the criteria for the types of CPD activities that could be considered acceptable for chiropractors were relaxed. A more flexible approach has been adopted which allows a practitioner to plan their CPD activities so that they meet the specified CPD goals and reflect on how they will improve their practice based on what they have learned [47]. Given the influence of heterogeneity between chiropractic schools regarding professional identity and scope of practice in pre-professional training [38], a high degree of flexibility in the post-professional area has the potential to promote further disparity [48].

### Professional identity

Although a clear understanding of professional identity is paramount for the progression of any healthcare profession [12], the problem of conflicting professional identity is well reported in the chiropractic literature [39]. However, since its inception, professional identity within chiropractic has been a source of controversy [48, 49]. Attempts to establish a single professional identity for the profession have been met with resistance based on disagreements over terminology [50], philosophy (e.g. approach to care) [51], technique (e.g. intervention) [52], and the role of chiropractic within the broader healthcare framework [53].

The Australian profession continues to be beleaguered by more than one intra-professional group, with each asserting differing views on identity [45]. For example, the majority of chiropractors identify with, and practice within an evidence-based ‘biopsychosocial’ model of care [54]. This is in contrast to a vocal minority of chiropractors who identify with a historical ‘vitalistic’ model of care which is based around diagnosis of the ‘vertebral subluxation’ [45], a concept unique to the chiropractic profession. This diversity appears to exist across chiropractic educational institutions [38, 45]. This situation has been exacerbated by some within the profession seeking to operate outside the accepted framework [45, 55]. As a result, the chiropractic profession has failed to establish a clear professional identity due primarily to the absence of a formulated scope of practice [49].

Simply stated, scope of practice is in part defined by whether chiropractors model their behaviour on a ‘subluxation-based’ model, where misaligned vertebrae are viewed as the cause of disease, or on an ‘evidence-based’ model where decision making around the diagnosis and management is based on current best evidence [38]. The situation is further complicated where there is direct evidence of efficacy and/or biological plausibility around the effect of one, but not the other [38].

The lack of clarity regarding professional identity was one of the drivers for developing accreditation standards that acknowledge both models [56]. It has been suggested that chiropractic educational institutions and the national accrediting body (CCEA) review their procedures for evaluating program curricula and accreditation standards to create a consistent standard across institutions, a strategy that may alleviate the incongruity that exists among students regarding professional identity [38].

Confusion around professional identity within the chiropractic profession is well reported in the literature [38]. This circumstance is contrary to other health professions. For example, in nursing, professional identity is clearly defined as a sense of oneself that is ‘influenced by the characteristics, norms and values of the discipline, resulting in the individual thinking, acting and feeling like a nurse’ [57]. The lack of clarity regarding chiropractic’s professional identity may be driving recent concerns raised by government [58], and the media [25], around patient safety.

### Patient safety

Patient safety aims to prevent injury, and reduce risks, errors and harm that can occur to a patient during the provision of a healthcare service. This is true for all healthcare professions, including those that use spinal manipulation as a therapeutic intervention, particularly in the field of paediatrics [13, 25, 58].

At first glance, it may not be easy to see how patient safety influences scope of practice, but international and Australian data help to clarify the issue. For example, the Nursing and Midwifery Board of Ireland [59] asserts that an “individual nurse’s scope of practice [the ‘personal scope’ mentioned previously] is dynamic, and is influenced by a number of factors including patient safety, patient needs, and care outcomes”. Additionally, the Federation of State Medical Boards of the United States contends that the concept of “patient safety should be considered by health care regulatory boards and legislative bodies when making decisions about changes in scope of practice” [60].

Furthermore, a small Australian study undertaken by Engel et al. [41], found that personal scope of practice of chiropractors can be negatively influenced by a perceived notion amongst surveyed GPs that chiropractic is unsafe. As GPs are a primary contact point for many MSK presentations, negative views of the chiropractic profession could translate into reduced referrals (number and type) from GP’s to chiropractors.

### Organisational structures

Healthcare organisational structures (e.g. a hospital) are social systems purposely designed for the delivery of necessary healthcare services by specialised workforces within the system [61–63]. When the structure, or more correctly the health professionals within the structure, are unable to provide suitable care, it is the responsibility of those within the structure to co-ordinate care from other providers outside of the primary care setting [64]. However, Netto et al’s. study [42] highlighted that Royal Australian Air Force (RAAF) physicians showed little interest in referring fighter jet pilots who commonly suffered from acute or chronic neck pain from aerial manoeuvres, for chiropractic treatment, until the on-base medical care had failed, a situation that potentially limited chiropractors from utilising and/or further developing their scope of practice.

### Limitations

There are several limitations associated with this study. It is possible that some articles may have been missed or excluded due to the chosen search parameters. Additionally, while relevant journal databases and grey literature were searched extensively, the number of included studies was small and varied in both aim and methodology. Therefore, the factors identified within this review may be incomplete.

### Future research

As innovative technology and new treatment modalities emerge, the importance of understanding scope of practice is increasing for health care professions, as they are called upon to navigate the increasingly complex realm of patient care. This is true for chiropractic. Although this review was limited to the scope of practice of chiropractic in Australia, we recognise that a broader picture exists across the spectrum of health professions in the country. Future research could address whether the regulated professions function better without a defined scope of practice as in medical practice, or whether the approach seen in dentistry with a formal, defined scope of practice is more acceptable for a profession and its patients. Such studies may help determine if it is necessary to have a common defined scope of practice for the various professions. This would assist in determining the best framework for guaranteeing competency and patient safety.

## Conclusion

This scoping review investigated the current state of literature on factors that influence scope of practice of chiropractic in Australia. Four factors were found: education, professional identity, and organisational structures. Recognising these factors in the literature provides the foundation for identifying what is acceptable in the profession, and the role chiropractic has in the broader healthcare system. Understanding these factors will help inform future discussions between stakeholders such as educators, employers, consumers, funding bodies, policy makers, and practitioners around establishing a readily acceptable scope of practice of chiropractic in Australia. Such a framework has the potential to benefit patient safety, professional identity, public perception, education, and regulation of the profession.

## Supplementary Information

**Additional file 1: Appendix 1.** Samples of database search strategy.

**Additional file 1: Appendix 2.** Overview of included studies.

## Data Availability

All data generated or analysed during this study are included in this published article.

## Abbreviations

ABC: Australian Broadcasting Corporation
AHPRA: Australian Health Practitioner Regulation Agency
CBA: Chiropractic Board of Australia
CCEA: Council on chiropractic education of Australasia
CPD: Continuing professional development
GP: General practitioner
JBI: Joanna Briggs Institute
MSK: Musculoskeletal
OSF: Open science framework
PCC: Population, concept, context
PRISMA-ScR: Preferred reporting items for systematic reviews and meta-analyses extension for scoping reviews
SMT: Spinal manipulative therapy
WHO: World Health Organization

## Declarations

### Review protocol

There is no published protocol document for this research.
