# Supplementary material for: Factors that influence scope of practice of the chiropractic profession in Australia: a scoping review
Source: Chiropr Man Therap. 2022 Apr 14;30:19. doi: 10.1186/s12998-022-00428-2 (PMC9011944; doi:10.1186/s12998-022-00428-2)
Supplement: Supplementary file 1 — Additional file 1: Appendix 1. Samples of database search strategy. [file 12998_2022_428_MOESM1_ESM.docx]

**Additional file 1: Appendix 1:** Samples of database search strategy

**MEDLINE (EBSCO) Search Strategy**

1. Chiropractic or chiropractor

2. Scope of practice

3. Determinant/s (or barrier/s or influencers or facilitators) of scope of practice

4. Australia

5. 1 AND 2

6. 1 AND 3

7. 1 AND 2 AND 4

8. 1 AND 2 AND 3 AND 4

**CINAHL (EBSCO) Search Strategy**

1. Chiropractic or chiropractor

2. Scope of practice

3. Determinant/s (or barrier/s or influencers or facilitators) of scope of practice

4. Australia

5. 1 AND 2

6. 1 AND 3

7. 1 AND 2 AND 4

8. 1 AND 2 AND 3 AND 4
