# Supplementary material for: Factors that influence scope of practice of the chiropractic profession in Australia: a scoping review
Source: Chiropr Man Therap. 2022 Apr 14;30:19. doi: 10.1186/s12998-022-00428-2 (PMC9011944; doi:10.1186/s12998-022-00428-2)
Supplement: Supplementary file 2 — Additional file 1: Appendix 2. Overview of included studies. [file 12998_2022_428_MOESM2_ESM.docx]

**Additional file 2: Appendix 2:** Overview of included studies

| Category | Year | Author/s | Country of Origin | Aim/Purpose | Method/Study Design | Determinants |
| --- | --- | --- | --- | --- | --- | --- |
| Chiropractic | 2016 | Australian Broadcasting  Corporation [31] | Australia | To highlight the negative impact of paediatric chiropractic | News item | 1) Patient safety |
| Chiropractic | 2016 | McArthur, G. [17] | Australia | To highlight the negative impact of paediatric chiropractic | News item | 1) Patient safety |
| Chiropractic | 2016 | Engel et.al*.* [32] | Australia | To determine the current views of GPs towards chiropractic in Australia | Cross-sectional study | 1) GP perceptions influence  referral patterns |
| Chiropractic | 2018 | Innes et.al. [30] | Australia | To explore the link between chiropractic education and scope of practice | Qualitative study | 1) Level of chiropractic  education |
| Chiropractic | 2018 | de Luca et.al. [29] | Australia | To evaluate Australian and New Zealand chiropractic students’ opinions regarding the identity, role setting, and future of chiropractic practice | Cross-sectional  survey | 1) The educational institution  influences the scope of  practice of graduates |
| Chiropractic | 2011 | Netto et. al. [33] | Australia | To synthesise published literature exploring the nature, models, and outcomes of chiropractic services provided to active duty military globally | Scoping review | 1) System/facility *i.e.* the rules  and regulations of the base  hospital regarding  chiropractic treatment |
